# Occupational Stress: Preventing Suffering, Enhancing Wellbeing ^†^

**DOI:** 10.3390/ijerph13050459

**Published:** 2016-04-29

**Authors:** James Campbell Quick, Demetria F. Henderson

**Affiliations:** 1 Department of Management, The University of Texas at Arlington, Box 19377, Arlington, TX 76019, USA; 2 Faculty of Humanities, University of Manchester, Manchester M15 6PB, UK; 3 Department of Management, The University of Texas at Arlington, Box 19467, Arlington, TX 76019, USA; henderson@uta.edu

**Keywords:** occupational stress, occupational health, prevention, preventive stress management, stress, wellbeing

## Abstract

Occupational stress is a known health risk for a range of psychological, behavioral, and medical disorders and diseases. Organizations and individuals can mitigate these disorders through preventive stress management and enhanced wellbeing. This article addresses, first, the known health risk evidence related to occupational stress; second, the use of preventive stress management in organizations as the framework for intervention; and third, the emerging domain of enhancing wellbeing, which strengthens the individual. Premature death and disability along with chronic suffering from occupational stress are not inevitable, despite being known outcome risks.

## 1. Introduction

Stress is a rubric for the causes (demands or stressors), consequences (distress and eustress), and modifiers of the psychophysiological phenomenon known as the stress response. Cannon [1] coined “the emergency response” as the label for the complex of mind-body actions now known as the stress response. Selye [2] demonstrated how stress is a risk factor for a range of health disorders and diseases, which he labeled diseases of maladaptation. Quick and Quick [3] brought the public health notions of prevention into an organizational stress context, forging the theory of preventive stress management. Occupational stress was identified during the 1980s as one of the top ten occupational health problems in the United States and likely throughout the Western industrialized nations. Sauter, Murphy, and Hurrell [4] began developing a prevention agenda for addressing what some called an epidemic of stress.

Stress is directly linked to seven of the ten leading causes of death in the world, with cardiovascular disease being *the* leading cause for both men and women [5]. Occupational and organizational stress is a key cardiovascular risk factor [6]. Despite the fact that occupational stress is a known risk factor for health disorders, a positive side of the issue is found in notions from public health and preventive medicine. Our purpose is to briefly review the known health risk evidence regarding occupational stress, then incorporate enhancing wellbeing and positive psychology with the known theory of preventive stress management.

We achieve this purpose through three major topics of the article. The first topic briefly reviews the known health risk evidence related to occupational stress, covered in Section 1, Section 2, Section 3, Section 4, Section 5, Section 6 and Section 7. The second topic presents preventive stress management in organizations as the framework for intervention, covered in Section 8, Section 9, Section 10 and Section 11. The third topic extends the prevention framework with the emerging domain of enhancing wellbeing and positive psychology, covered in Section 12. Premature death and disability along with chronic suffering from occupational stress are not inevitable despite being known outcome risks.

## 2. Occupational and Organizational Stress

To call occupational stress a risk factor requires consideration of the life history of the problem. Occupational stress is not an acute or toxic condition that can be cured through treatment. Rather, it is a chronic condition that requires an understanding of the epidemiology or life history of the problem prior to exploring protection, prevention, and intervention alternatives. The epidemiology of occupational stress may be considered in three stages:

*Stage 1* includes the causes of stress, which are known to be risk factors.
*Stage 2* is the stress response, a normal and naturally occurring reaction to environmental demands or internal pressures.
*Stage 3* includes the consequences of the life history—either forms of distress (medical, psychological, behavioral) or forms of eustress (healthy stress).

In addition to the main stem of the life history of occupational stress, the stress response manifests a number of individual difference modifiers, which may either serve as protection factors for the individual or further open vulnerability. Because stress is not a specific disorder, it tends to attack a weak point, also known as one’s Achilles heel. This is sometimes called the weak organ hypothesis.

## 3. Causes of Stress: Demands and Stressors

A broad set of occupational and work demands as well as environmental stressors can trigger the stress response. While pressures may vary, some occupational stress concerns span occupations. Work-family conflict is one of those overarching risks, as demands from the home and for personal space tumble into the workplace. Hammer and her colleagues [7] addressed both the family-to-work and work-to-family conflicts. From Adolf Meyer’s early development of life charts to study health disorders, Holmes and Rahe [8] developed a systematic approach for understanding how life change events, such as financial problems or the death of a spouse, increase health risks for the individual [9].

Cooper and Payne [10] charted one of the original overviews for understanding stress at work by attending to epidemiology of work stress, both blue-collar and white-collar stressors, and the family as a cause of stress. Quick, Quick, Nelson, and Hurrell [11] provided an exhaustive review of the research literature and identified four broad categories of workplace demands—task demands (occupation, careers, workload, job insecurity); role demands (role conflict and ambiguity); physical demands (temperature, lighting, workplace design); and interpersonal demands (social density, personality conflicts, leadership style, group pressures).

When examining the causes of occupational stress, three sets of demands command our attention—occupational and work, home-based or family, and individual generated or internally imposed. The lack of employee decision latitude [12] is especially problematic in high-strain jobs, that is, high work demands and low control [13]. The second leading cause is uncertainty about any number of workplace aspects. Job insecurity, questions about the consequences of one’s actions, *etc.*, create chronic allostatic load [14], which in the long run brings physical wear and tear. The third leading cause of stress is poorly managed conflict at work [15]. Not all conflict is bad, of course; conflict that resolves issues is both constructive and functional.

## 4. The Stress Response

Cannon’s [16] seminal research on the physiological impact of emotions such as fear and rage established the fundamental architecture of the stress response. He determined that perception and emotion trigger the release of catecholamines by the adrenal glands, primarily epinephrine and norepinephrine. Catecholamine release has a direct, activating effect on the central nervous system and, in particular, the reticular activating system (RAS), or reticular formation. RAS stimulation leads to the alert state that usually occurs under stress.

While fight or flight may characterize the primary physiological response to stress in both genders, Taylor and her colleagues [17] made a major advance in the understanding of females’ responses to stress. Specifically, they found that behaviorally females are more marked by a pattern of “tend and befriend”. This life-giving response is likely one element accounting for females outperforming males under stressful conditions and thus outliving males by five to seven years. In addition to this gender difference, Lundberg [18] found that women often perform repetitive tasks and are more exposed to stress from unpaid work, which can lead to more work-related upper extremity disorders.

## 5. Individual Differences

We conclude that everyone exhibits a systemic and patterned psychophysiological stress response, with some gender difference. However, there are individual difference factors that play a role in either elevating vulnerability to occupational stress or protecting against the risks associated with it.

### 5.1. Vulnerability

At least three individual differences increase vulnerability. One is lower socioeconomic status, which exposes a person to a range of diseases and causes for mortality [19]. A second is coronary-prone, or Type A, behavior, a complex of competitiveness, quantification of achievements, time urgency, and hostility [20], often with hostility or anger the lethal component. The third individual difference is social isolation or loneliness [21].

### 5.2. Protection

There is good news, too, regarding individual differences. The protective factor of self-reliance characterizes strong interdependent relationships with others, which can bring about a health attachment/care-giving system [22,23]. In addition to protection, the secure relationships are a source of information, evaluative feedback, instrumental support, and emotional strength. Then there’s personality hardiness, characterized by commitment, control, and challenge, which provides protection as a stress-resistance variable [24].

## 6. Distress: Unhealthy Consequences

Distress is the unhealthy consequence of occupational stress and may result from prolonged, frequent, or intense stress exposure. Distress may occur in one of three forms—medical, psychological, or behavioral.

### 6.1. Medical Distress

The burden of medical distress comes primarily from heart disease, cancers, and musculoskeletal injuries with their associated pain and disability. Job stress has been identified as a major factor in cardiovascular disease. Landsbergis and his colleagues [25] linked cardiovascular disease to working conditions such as social isolation, shift work, chemicals, and physical hazards. Recent research by Baum, Trevino, and Dougall [26] portrays stress as causal to the onset of cancers and having an indirect role in worsening the disease and limiting recovery. Injuries are the fourth leading cause of death, while occupational injuries and associated pain can lead to significant work absences [27].

### 6.2. Psychological Distress

Anxiety and depression are the two lead presenting complaints associated with stress. Epidemiological research found a significantly high (nearly 10%) occurrence of mood and anxiety disorders within the U.S. population [28]. Gutman and Nemeroff [29] conclude that depression ranks among the largest contributors of morbidity and lost productivity. Depression will affect about 16% of adults. Burnout is a third significant form of psychological distress. Maslach [30] was the psychologist who first fully elaborated the burnout syndrome with its associated costs and suffering. She characterized burnout as emotional exhaustion, depersonalization or cynicism, and lack of personal accomplishment.

### 6.3. Behavioral Distress

The leading forms of behavioral distress include tobacco abuse, alcohol and drug abuse, aggression and violence, and accidents. The direct costs of smoking were pegged at $96 billion from 2001 to 2004 with an additional equivalent cost from lost productivity and increased health care expenditures [31]. Beyond tobacco abuse, alcohol use and impairment are highly prevalent within the U.S. workforce with 73.6% using alcohol, 30.6% becoming intoxicated, and 22.6% experiencing a hangover [32]. Alcohol abuse affects attendance, task performance, accidents, and injuries. Drug abuse had an annual cost of $180.9 billion in 2002 and included lost productivity from disability, death, and withdrawal from the workforce [33]. O’Leary-Kelly, Griffin [34] brought attention to workplace aggression and violence, which increased dramatically in the decade 1997–2007. Finally, industrial accidents cause distress and are the leading cause of death for males in the workplace [35].

## 7. Eustress: Good Outcomes

Since 2000 the literature has shown an increased appreciation of the positive consequences of stress. Eustress is good stress, from the medical prefix eu. The Nelson and Simmons [36] model of eustress, with its in-depth assessment of hope, positive affect, vigor, meaningfulness, manageability, satisfaction, and commitment, is a major contribution. Savoring eustress is a key element in the model. Using a different approach, Lepine, Podsakoff, and Lepine [37] move the agenda forward by spotlighting challenge stress, or good stress. Challenge stressors help people grow and become more competent.

## 8. Preventive Stress Management ^TM^

Quick and Quick [3,38] introduced the theory of preventive stress management (TPSM) when they translated the public health notions of prevention into an occupational or organizational context. A 33-year review of TPSM demonstrated how it has contributed to theoretical understanding, empirical exploration, and organizational practices [39]. The authors extended the original theory as presented in Cooper [40] by adding a Corollary following Hypothesis 3 that focuses on the individual differences discussed above. The Corollary is particularly important as we extend the prevention framework in our discussion of wellbeing:

*Hypothesis 3*: Individuals high in vulnerability modifiers are at greater risk of distress than individuals low in vulnerability modifiers.
*Corollary*: Individuals high in protective mechanisms and defenses are shielded from the risk of distress more than individuals low in these factors.

Protection and prevention are at the heart of a public health response to occupational stress. In this section we address both levels (organizational and individual) and all three stages (primary, secondary, tertiary) of preventive stress management. We then extend the prevention framework in our discussion of enhancing wellbeing and positive psychology.

## 9. Preventive Medicine and Occupational Health

By using preventive medicine and epidemiology as a launch point while adding contributions from psychology (especially occupational health psychology) and engineering, a more positive future for occupational health is easily conceived [41]. Because occupational stress is a chronic issue, eradication can never be the goal. But all occupational stress is not toxic. Therefore, the preventive management concepts from public health lend themselves nicely to the regulation of occupational stress, bringing out the positives and minimizing the suffering caused by distress.

## 10. Organizational Protection and Leadership

Defense against the adverse outcomes of occupational stress begins at the organizational level. An organization that is committed to protecting people in reasonable and appropriate ways is investing in the future. Organizational protection and prevention is especially important because it addresses the Stage 1 workplace stressors and risk factors, aiming to change the environment. Work stress prevention in Europe, including the United Kingdom, places the emphasis on organizational protection, and the law itself sets standards in this regard [42]. Even in the United States there is an emerging priority on organizational responsibility regarding occupational stress and health.

Adkins [43] framed occupational health as a leadership challenge and crafted an approach to promoting it. From her pioneering work came the organizational health center as a comprehensive, multidisciplinary, multi-component system designed to implement key elements of public health, preventive medicine, and health protection. Klunder [44] implemented the concept in a six-year intervention within a 13,000-person aerospace logistics and maintenance depot with direct support from the commanding general officer. An organizational health center has three distinguishing characteristics:

1. Collaboration among all organizational functions concerned with people’s wellbeing
2. Surveillance systems for early warning of distress and dysfunction
3. The full continuum of protection and prevention interventions to benefit everyone in the organization

The results of Klunder’s organizational health center intervention work were: (1) zero fatalities through suicide or homicide; (2) zero incidences of workplace violence; and (3) over U.S. $33 million in cost avoidance from complaints that failed to materialize according to the HR staff.

Regardless of how much organizational protection leaders provide, occupational stress will have different impacts throughout a working population. Therefore, a supplemental need exists for individual preventive stress management. Individuals may use several tools to lessen the stresses and strains of the workplace.

## 11. Preventive Stress Management for Individuals

Despite the best organizational programs to rally the work environment, individual differences call for supplementary individual skills. Preventive stress management for individuals offers such skills through three sets of interventions that aim at countering the sources of stress (primary prevention), alter the responsibility to inevitable stress (secondary prevention), and help alleviate any suffering that may arise (tertiary prevention). One or two prevention tools are probably insufficient for an individual. Rather, the greatest benefits come from using the prevention methods that are most appropriate for a specific person and her/his circumstances.

*Primary prevention* is stressor directed and aims to help the individual manage personal perceptions of stress, elevate her/his personal work environment, and maintain work-life balance. While a range of techniques can help achieve the best outcomes, two of the leading primary prevention tools are appropriate positivity ration and good social supports. Fredrickson [45] suggests that individuals flourish when their positive feelings outweigh their negative feelings. Social support is a robust primary prevention intervention for occupational stress, and Lyubomirsky [46] provides recommendations for creating positive relationships.

*Secondary prevention* is response directed and aims to help the individual regulate stress-induced energy, emotions, and physical fitness. Physical fitness programs have become widely available to employees as a way to prevent distress [47]. The relaxation response continues to be a time-honored stress antidote [48].

*Tertiary prevention* is symptom directed and draws on treatment therapies and counseling interventions to restore health and function. While a number of psychological and medical treatment interventions may help individuals suffering from occupational stress, emphasizing emotional health in the workplace should be a priority for organizations and occupations [49].

## 12. From Stress Prevention to Enhancing Wellbeing

An area of research germane to occupational stress is the burgeoning field of workplace health and wellbeing. Individuals today are on a quest for happiness, meaningfulness, and inner peace, yet many find this pursuit challenging due to stress at work and home. Occupational stress can have far-reaching consequences. In their stress model emphasizing the relationship between the sources of work-related stress (intrinsic job characteristics, home-work interface, interpersonal work relationships), Cooper and Cartwright [50] argue that occupational stress can detrimentally affect the body and mind of employees as well as a firm’s financial health. Consider the home-work interface, in which people experience increased stress as their work lives overpower their non-work lives, and *vice versa* [51]. One encouraging avenue of research is the wellbeing concept, including positive psychology. We suggest this extends from the prevention framework, enhancing TPSM.

### 12.1. Introducing Wellbeing and Positive Psychology

Wellbeing is defined as “peoples’ positive evaluations of their lives, (which) includes positive emotion, engagement, satisfaction, and meaning” [52]. Although this definition may appear simple, wellbeing is in fact a complex construct with two distinct approaches: the hedonic and the eudaimonic [53]. The hedonic view, also called subjective wellbeing, focuses on happiness. Researchers using the hedonic perspective seek to understand the conditions and processes for attaining pleasure and avoiding pain. The eudaimonic approach (*i.e.*, psychological wellbeing), on the other hand, centers on meaning and self-realization and conceptualizes wellbeing as a person’s ability to fully function [53].

Positive psychology and TPSM offer a good framework for studying wellbeing. Positive psychology comprises the processes that lead to flourishing and optimal functioning [54] as well as the study of positive emotions and character traits and their enabling factors, [55] which can be useful for the prevention and management of stress. Ultimately, positive psychology shifts the narrative from a *need to fix* to a mind-set that builds on positive qualities that capture the full spectrum of the human experience [54,56]. The introduction of positivity to the stress literature is not a novel idea, as referenced by our earlier discussion of eustress. In fact, in recent years, Nelson and Cooper [57] have argued for more researchers to focus on traits that promote good health and wellbeing as opposed to bad health and negative consequences.

At the individual level, positive psychology examines positive traits such as courage, perseverance, spirituality, wisdom, and forgFiveness; the group level highlights civic virtues, social support, tolerance, and other factors that draw individuals to engage in more responsible ventures and acts of citizenship [54,56]. Measurements of wellbeing often utilize the Satisfaction With Life Scale [58]. Coincidentally, this emphasis aligns with positive psychology’s objective to study the three aspects of life: the pleasant life; the good life; and the meaningful life [59].

Park, Peterson, and Seligman [60] found that the character strengths of zest, hope, love, and gratitude result in higher life satisfaction. They assert that fulfillment is not the result of virtuous action but a necessary component of our actions [60]. This finding implies that developing these characteristics through interventions becomes inherent, resulting in greater life satisfaction [60].

### 12.2. Positive Psychology Interventions

Positive psychology interventions (PPIs) stem from research that focuses on teaching individuals how to increase positive thinking and behaviors through brief, self-administered exercises [61,62]. Utilizing the theory of preventive stress management, PPIs can be administered at the primary, secondary, and tertiary levels of preventive stress. In addition, they can function at both the individual and organizational levels. Examples of PPIs are writing gratitude letters, thinking optimistically, reimagining positive experiences, and socializing.

At the primary prevention level, PPIs focused on building strong, supportive social networks can be instrumental in coping with stress. Cohen and Wills [63] found that social support buffers stress, which contributes to greater wellbeing. Mentoring, building solid family relationships, and establishing cohesive networks with colleagues are a few interventions available to combat stressors in the workplace. At the organizational level, goal setting interventions, which include career path planning and heightened role clarity, help organizations reduce stress [38]. In the United Kingdom, the Management Standards cut potential risk by ensuring that employees, middle management and top management all work together in risk assessment processes such as job redesign and skill enhancement initiatives [64].

Secondary prevention PPIs include life coaching and mindfulness training, as well as education and development programs that build character strengths. Berg and Karlsen [65] argue that coaching intervention plans, including positive visualization and self-talk, give employees the means to cope with work pressures. Organizational policies promoting team building and a strong supportive supervisor-employee relationship have also been shown to protect employees from the adverse effects of stress [66,67].

At the tertiary prevention level, interventions are designed to heal. PPIs here may include therapy, medical assistance, and meditation. For organizations, PPIs include development interventions and wellness programs as a means for healing the negative consequences of distress and strain.

In sum, utilizing PPIs in health and wellbeing research may prove to be a low-cost, effective approach in ameliorating the effects of occupational stress. Danna and Griffin [51] developed a framework for research on workplace health and wellbeing in which they advocated for interventions to help improve workplace safety, reduce occupational stressors, and improve coping skills. Fordyce [68,69] showed that behavioral programs—interventions designed as a series of exercises modeled from the characteristics of happy people—have a positive effect on personal contentment. Fordyce’s findings suggest that happiness increased through interventions. In a meta-analysis of positive psychology interventions, Sin and Lyubomirsky [62] found that PPIs have a significant effect on increasing wellbeing.

### 12.3. Promotion of Wellbeing and Health

A synthetic analysis of 30 follow-up studies on happiness and longevity found that happiness failed to predict longevity in sick populations, but it did among healthy populations [70]. Veenhoven [70] says that “we can make people healthier by making them happier” [70]. The implication is not that happiness cures illness but, more importantly, that it serves as a protective factor against becoming ill. As a result of his work, Veenhoven [70] suggests four probable ways how wellbeing, or happiness, promotes health:

1. Chronic unhappiness triggers the fight-or-flight response, resulting in higher blood pressure and a lower immune defense. Cohen, *et al.* [71] found that negative affect is significantly related to increased health complaints.
2. Happy people are more inclined to engage in healthy behaviors, such as monitoring their weight and alcoholic intake as well as being more physically active.
3. Happy people have a broader array of resources such as a strong social support system they can draw upon in times of stress. Argyle [72] found that relationships have a significant influence on the immune system, resulting in improved health.
4. Happy people make better life choices and are more prone to avoid distress.

To summarize, increasing wellbeing through the use of PPIs positively influences mental and physical health outcomes. Interventions are relatively low-cost options to combat the adverse effects of occupational stress; they give individuals the personal resources they need to become productive, healthy employees. Positive psychology interventions used in conjunction with the theory of preventive stress management is a promising avenue that practitioners and researchers should pursue for enhancing wellbeing.

## 13. Conclusions

Occupational stress is an inevitable, even at times necessary, element of the work environment, but it does not have to translate into organizational dysfunction nor medical, psychological, or behavioral distress. We briefly reviewed the evidence concerning the health risks associated with occupational stress, then focused on the application of preventive stress management, developed from roots in preventive medicine and public health, to enhance eustress and avert distress.

We emphasize that organizational protection and work environment prevention come first in managing occupational stress but must be supplemented because of individual differences. Therefore, we move from stress prevention to enhancing wellbeing by introducing positive psychology to broaden the TPSM framework. Addressing organizational protection, prevention, and individual wellbeing raises the overall public health of the work environment. In addition, positive psychology skills for occupational stress may have positive spillover effects into the home.
